# Unleashing the potential of health data: from vision to reality in the HealthData@EU Pilot Project

**DOI:** 10.1093/eurpub/ckaf003

**Published:** 2025-09-10

**Authors:** Fulvia Raffaelli, Martin Dorazil, Mélodie Bernaux, Owe Langfeldt, David Asturiol, Jerome De Barros, Irini Kessissoglou, Licinio Kustra Mano, Guillaume Byk

**Affiliations:** European Commission, Directorate-General for Health and Food Safety, Unit C1—Digital Health, Bruxelles, Belgium; European Commission, Directorate-General for Health and Food Safety, Unit C1—Digital Health, Bruxelles, Belgium; European Commission, Directorate-General for Health and Food Safety, Unit C1—Digital Health, Bruxelles, Belgium; European Commission, Directorate-General for Health and Food Safety, Unit C1—Digital Health, Bruxelles, Belgium; European Commission, Directorate-General for Health and Food Safety, Unit C1—Digital Health, Bruxelles, Belgium; European Commission, Directorate-General for Health and Food Safety, Unit C1—Digital Health, Bruxelles, Belgium; European Commission, Directorate-General for Health and Food Safety, Unit C1—Digital Health, Bruxelles, Belgium; European Commission, Directorate-General for Health and Food Safety, Unit C1—Digital Health, Bruxelles, Belgium; European Commission, Directorate-General for Health and Food Safety, Unit C1—Digital Health, Bruxelles, Belgium

## Introduction: the untapped potential of health data

How can we unleash the full potential of health data to drive research, innovation, and public health improvements across Europe? This is the central question answered by the European Health Data Space (EHDS) regulation, a cornerstone of the EU’s data strategy. While the road towards the improved use of health data for primary use is well-established, its secondary use—for research, policymaking, and innovation—has long suffered from fragmented access, long delays, and a lack of common streamlined processes.

The HealthData@EU Pilot Project has been instrumental in creating building blocks to turn the vision of the EHDS into a reality. This editorial examines how the HealthData@EU Pilot Project has provided practical solutions to shape the future of health data governance.

## The genesis of the EHDS2 framework: a strategic response to a critical need

The EHDS was conceived to address a pressing need for a unified, accessible health data infrastructure across the EU. The COVID-19 pandemic exposed the limitations of fragmented national systems and underscored the importance of seamless, cross-border data sharing for effective public health responses. It was clear that Europe required a cohesive common framework to fully leverage health data for research, innovation, and policy purposes.

However, creating this common framework presented significant challenges due to the Member State’s diverse regulatory, health environments, and varying levels of digital maturity.

The first TEHDAS Joint Action (Joint Action Towards the European Health Data Space—TEHDAS1—Tehdas, https://tehdas.eu/tehdas1/) project was a key early initiative that identified barriers to the secondary use of health data across the EU. These barriers included inconsistent access rules, differing legal frameworks, and incompatible data formats (lack of interoperability) between Member States. The first TEHDAS Joint Action played an essential role in laying the groundwork for the regulation by advocating for common standards, infrastructures, and proposing a unified approach to make cross-border data use more feasible.

## The HealthData@EU Pilot Project: a pragmatic approach

How do we move from vision to a functional, pan-European health data space? The HealthData@EU Pilot Project sought to answer this question by developing and testing solutions that could bridge the gaps identified in first TEHDAS Joint Action. The pilot tested regulatory and technical solutions in use-cases—real-world scenarios, offering concrete examples that informed policy discussions and helped resolve key questions.

Among its key achievements was the creation of the Common Application Form, which provides a standardized template for requesting access to health data across all Member States. The form simplifies and streamlines the application process. At the same time, the regulation establishes a harmonized vetting and approval procedures that ensure the safeguarding of privacy and the sensitive nature of health data. By demonstrating how to implement the future legal framework in a practical and consistent manner, the Common Application Form contributes to building the trust necessary for Member States and citizens to engage fully in the EHDS initiative.

The pilot also introduced Health DCAT-AP (Health Data Catalogue Application Profile), a standardized metadata framework designed to enhance the discoverability and ensure interoperability of health datasets’ descriptions across Europe. By establishing common open metadata standards, the pilot enables the federation of dataset descriptions in a single central EU portal, what increases the discoverability and findability of health data across the EU, meaning that researchers and public health officials can more easily understand what data are available and identify the most relevant datasets for their projects, regardless of where the data are stored.

Additionally, the HealthData@EU Pilot Project provided input on economic scenarios and offered recommendations for establishing Health Data Access Bodies and national nodes. These contributions support the creation of a sustainable framework that can accommodate a wide range of uses, from public health monitoring to cutting-edge research and artificial intelligence.

Finally, the HealthData@EU Pilot Project underscored the importance of dialogue and collaboration among diverse stakeholders. Researchers, IT specialists, policymakers, legal experts, and metadata professionals all contributed to the project’s success. Their collective efforts advanced the technical aspects of the EHDS, while ensuring the system was designed to meet the practical needs of end-users, including healthcare providers and public health authorities.

## Impact and future directions: from vision to practice

The HealthData@EU Pilot Project has demonstrated that a unified, efficient, and secure health data infrastructure is not only desirable but also achievable. By addressing the technical barriers to the secondary use of health data and offering practical solutions to help shape the legal framework, the pilot has proven that the EHDS vision can become a reality.

As Europe continues to build on the foundation laid by the HealthData@EU Pilot Project, these solutions will serve as a blueprint for future initiatives. The project’s success is a testament to the power of collaboration across Europe, demonstrating that when Member States work together, they can overcome complex challenges and create a unified common health data space that benefits us all.

## Conclusion: a pledge to public health and innovation

The HealthData@EU Pilot Project marks a significant milestone in the journey from the initial concept of the EHDS to its impending full-scale implementation. By addressing many of the challenges associated with creating a Europe-wide health data infrastructure, the project has provided valuable insights and tools that will guide the next stages of development.

As we look to the future, the lessons learned from the pilot will be instrumental in ensuring that the EHDS delivers on its promise of improving public health through better data access and sharing across the EU. The commitment to advancing this initiative remains strong, and the potential benefits for public health in Europe are immense.

